# Prospective Longitudinal Perfusion in Probable Alzheimer’s Disease Correlated with Atrophy in Temporal Lobe

**DOI:** 10.14336/AD.2023.0430

**Published:** 2024-08-01

**Authors:** Tony D Zhou, Zongpai Zhang, Arvind Balachandrasekaran, Cyrus A Raji, James T Becker, Lewis H Kuller, Yulin Ge, Oscar L Lopez, Weiying Dai, H. Michael Gach

**Affiliations:** ^1^Department of Radiation Oncology, Washington University School of Medicine, Saint Louis, MO 63110, USA.; ^2^Computer Science, State University of New York at Binghamton, Binghamton, NY 13902, USA.; ^3^Harvard Medical School/Boston Children’s Hospital, Boston, MA 02115, USA.; ^4^Departments of Radiology and Neurology, Washington University School of Medicine, Saint Louis, MO 63110, USA.; ^5^Departments of Psychiatry, Psychology, and Neurology, University of Pittsburgh, Pittsburgh, PA 15260, USA.; ^6^Department of Epidemiology, University of Pittsburgh, Pittsburgh, PA 15213, USA.; ^7^Department of Radiology, New York University School of Medicine, New York, NY 10016, USA.; ^8^Departments of Neurology and Psychiatry, University of Pittsburgh, PA 15260, USA.; ^9^Departments of Radiology and Biomedical Engineering, Washington University in St. Louis, Saint Louis, MO 63110, USA.

**Keywords:** Alzheimer's disease, cerebral blood flow, arterial spin labeling, structural magnetic resonance imaging, gray matter volume

## Abstract

Reduced cerebral blood flow (CBF) in the temporoparietal region and gray matter volumes (GMVs) in the temporal lobe were previously reported in patients with mild cognitive impairment (MCI) and Alzheimer’s disease (AD). However, the temporal relationship between reductions in CBF and GMVs requires further investigation. This study sought to determine if reduced CBF is associated with reduced GMVs, or vice versa. Data came from 148 volunteers of the Cardiovascular Health Study Cognition Study (CHS-CS), including 58 normal controls (NC), 50 MCI, and 40 AD who had perfusion and structural MRIs during 2002-2003 (Time 2). Sixty-three of the 148 volunteers had follow-up perfusion and structural MRIs (Time 3). Forty out of the 63 volunteers received prior structural MRIs during 1997-1999 (Time 1). The relationships between GMVs and subsequent CBF changes, and between CBF and subsequent GMV changes were investigated. At Time 2, we observed smaller GMVs (p<0.05) in the temporal pole region in AD compared to NC and MCI. We also found associations between: (1) temporal pole GMVs at Time 2 and subsequent declines in CBF in this region (p=0.0014) and in the temporoparietal region (p=0.0032); (2) hippocampal GMVs at Time 2 and subsequent declines in CBF in the temporoparietal region (p=0.012); and (3) temporal pole CBF at Time 2 and subsequent changes in GMV in this region (p = 0.011). Therefore, hypoperfusion in the temporal pole may be an early event driving its atrophy. Perfusion declines in the temporoparietal and temporal pole follow atrophy in this temporal pole region.

## INTRODUCTION

Alzheimer’s disease (AD) is a neurodegenerative disease with progressive cognitive impairment, characterized by loss of neurons, plaques of insoluble amyloid-β(Aβ) and neurofibrillary tangles (NFTs) of phosphorylated microtubule-associated protein tau (P-tau) [1]. AD is associated with diminished cerebral blood flow (CBF) [2-4] and gray matter volume (GMV) [5-7]. Declines in CBF were related to the decline of cognitive function and may precede the appearance of the clinical syndrome by many years [8]. The most consistent finding in the literature for mild cognitive impairment (MCI) and AD is decreased CBF and metabolism in the temporoparietal region at the early stage based on single photon emission computed tomography (SPECT), perfusion MRI, and positron emission tomography (PET) [9-12]. However, gray matter atrophy in the medial temporal lobe (MTL) [13-15], including the hippocampus and entorhinal cortex, was predominantly reported. Dissociated regional patterns of CBF and GMV have been observed in these cohorts [13, 16]. Indeed, it has been debated whether decreased CBF precedes brain atrophy because of a pathological reduction in blood supply (such as small vessel diseases) or follows brain atrophy that induces reduced metabolic demand from neurodegeneration in AD.

One hypothesis proposed that hippocampal atrophy occurs earlier than hypoperfusion in the temporoparietal region [17]. Other studies proposed and supported that vascular damage and reduced perfusion in the parietal association cortex leads to the initiation and exacerbation of AD pathology in MTL [18, 19]. One recent cross-sectional study demonstrated significant association of MTL volumes and CBF values in the typically reduced-perfusion regions of AD, including the angular gyrus/temporoparietal gyrus, precuneus, posterior cingulate, and middle frontal cortex [20]. However, longitudinal studies are preferable for determining the temporal relationship between temporoparietal hypoperfusion and MTL atrophy.

This study took advantage of the longitudinal design of the Cardiovascular Health Study (CHS) Cognition Study (CHS-CS). CBF maps and GMV maps were derived from noninvasive arterial spin labeling (ASL) MRI and T_1_-weighted structural MRI, respectively. Voxel-based GMV maps were compared among normal cognitive (NC) control, MCI, and AD subjects. The AD-associated atrophic clusters in the voxel-based GMV comparisons were considered as the atrophy regions of interest (ROIs). We hypothesized that the AD-associated atrophy ROIs are located in MTL. The AD-associated CBF ROIs were derived previously from voxel-based CBF comparisons among NC, MCI, and AD subjects [21]. Our study focused on two ROIs: one AD-associated CBF ROI (the temporoparietal region) and one AD-associated atrophy ROI. We investigated whether the GMVs at Time 1 and Time 2 in each ROI were associated with subsequent CBF changes (from Time 2 to Time 3), or if the CBF values at Time 2 in each ROI were associated with subsequent GMV changes (from Time 2 to Time 3).

## MATERIALS AND METHODS

### Study population

The CHS is a multicenter study established in 1989 to investigate risk factors for cardiovascular disease in elderly individuals. Between 1997 and 1999 (Time 1), the CHS-CS recruited non-AD participants at the University of Pittsburgh and only high-resolution structural MRIs were acquired for the participants. Between 2002 and 2003 (Time 2), 195 participants received both structural and perfusion MRIs. Neuropsychological assessments and Modified Mini-Mental State Examination (3MSE) were performed at both Time 1 and Time 2 [22-24]. The subjects were classified as NC, MCI, or AD yearly based on cognitive status adjudications [25] without verification of positive Aβor positive tau. Therefore, AD should be viewed as probable AD herein. MCI, including MCI-amnestic and MCI-multiple cognitive domain types, was classified according to the CHS-CS diagnostic criteria [25]. The MCI amnestic type included subjects with impairments (defined as performance 1.5 SD below age/education appropriate means) in delayed recall of verbal or nonverbal material (or both) and with cognitive deficits that represented a decline from a previous level of functioning. Cognitive functions otherwise fell within normal limits. The diagnosis did not exclude individuals with mild alterations on instrumental activities of daily living (IADLs). Diagnosis of the second type, MCI-multiple cognitive deficits, required impairments in at least one cognitive domain other than memory (i.e., results of two or more tests were abnormal), or else one abnormal test result (which could be a memory test result) in at least two separate domains, without sufficient severity or loss of IADLs to constitute dementia. These cognitive deficits may or may not affect IADLs but must represent a decline from a previous level of functioning in order to fulfill the diagnostic criteria. Participants were classified with possible MCI when there were psychiatric, neurologic, or systemic conditions that could themselves cause cognitive deficits. Subjects were classified as having probable MCI when no comorbid factors were identified. A diagnosis of dementia was based on a deficit in performance in two or more cognitive domains that was of sufficient severity to affect IADLs, with a history of normal intellectual function before the onset of cognitive abnormalities. A memory deficit was not required for the diagnosis of dementia. The cognitive classification was interpolated based on the time of each MRI [26]. Prior inclusion and adjudication criteria were used [27].

**Figure 1.**
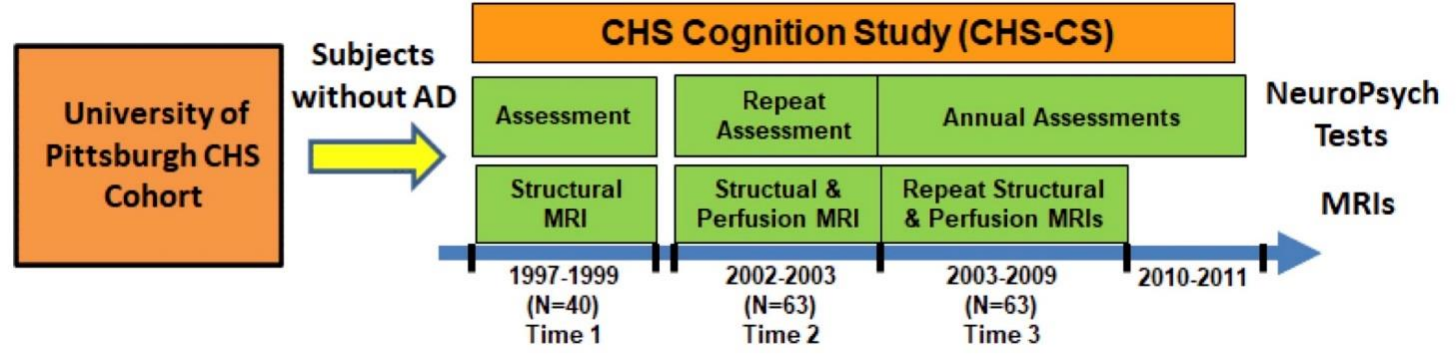
**Study schema**. Sixty-three volunteers from the Cardiovascular Health Study (CHS) Cognition Study (CHS-CS) were used for this study. All of the volunteers received two sets of structural and perfusion MRIs during 2002-2009. The 63 included 40 volunteers from the University of Pittsburgh cohort of the CHS who also received neuropsychological assessment and structural MRIs during 1997-1999. None of the volunteers had AD when they were recruited for the CHS-CS.

The detailed summary for excluded subjects was previously listed [21]. Based on the exclusion criteria, 148 participants, including 58 NC subjects, 50 MCI patients, and 40 AD patients, had follow-up structural and ASL perfusion MRIs at Time 2 from 2002 to 2003. Between 2003 and 2009 (Time 3), Sixty-three of the 148 participants, including 15 stable-NC, 14 NC-to-MCI, 16 stable-MCI, and 18 MCI/AD-to-AD (9 MCI-to-AD, 9 stable-AD), had follow-up structural and perfusion MRIs (Fig. 1). Structural MRI and perfusion MRI occurred at the same MRI scanning session at either follow-up times (Time 2 or Time 3) for each subject. To obtain prior longitudinal data, we looked for MRI scans of the 63 subjects in 1997-1999. Forty out of the 63 participants, including 37 NC subjects and 3 MCI subjects, had structural MRIs (Time 1). No perfusion MRIs were performed in 1997-1999. Details of the longitudinal data at the three time points are listed in Figure 1.

### Image acquisition

A GE Signa 1.5 T MRI (LX Version) at the University of Pittsburgh MR Research Center was used for all MRI scans. MRI images were acquired using a quadrature transceiver head coil. Written informed consent forms were approved by the institutional review board (IRB) and signed by all subjects or their caregivers. For high-resolution structural MRI, 3D T_1_-weighted spoiled gradient recalled echo (SPGR) structural images covering the whole brain were obtained (Voxel: 1x1x1.5 mm^3^, 5 ms echo time, 40° flip angle) [27, 28]. For perfusion MRI, labeling and acquisition sequences were multi-slice continuous arterial spin labeling (CASL) [27, 29, 30] and axial echo planar imaging (EPI). The MRI parameters were previously presented [30].

### Statistical analysis for demographic variables

Forty out of 63 subjects had earlier structural scans at Time 1 in 1997-1999. We tested whether the 23 subjects without earlier structural scans had demographic (age, gender, education, hypertension, diabetes, and heart disease) differences from the 40 subjects with earlier structural scans at Time 1. The age of each subject at Time 1 was calculated based on his/her scanned or scheduled MRI date. All tests were 2-tailed and a p-value less than 0.05 was considered statistically significant. The Shapiro-Wilk test was used to assess normality of variables. Differences of each demographic variable were evaluated using two-sample t-tests or Mann-Whitney nonparametric U-tests for continuous variables, and χ^2^ tests for categorical variables.

### Voxel-wise GMV analysis

#### Pre-processing

Voxel-based morphometry (VBM) in SPM12 (Statistical Parametric Mapping) was used to register structural MRIs of different subjects to a common template through spatial normalization [31]. However, conventional VBM suffered from imperfect registration to the standard brain [32]. Diffeomorphic Anatomical Registration Through Exponentiated Lie (DARTEL) was introduced to allow for precise image registration [33]. DARTEL-based VBM was reported to provide a greater diagnostic accuracy in AD than conventional VBM methods [34]. Structural MRI data from 148 image sets at Time 2, 63 follow-up image sets at Time 3, and 40 image sets at Time 1 were spatially normalized with DARTEL-based VBM with SPM12 software. First, segmentation was performed to generate gray matter and white matter images in native space and DARTEL-imported space. Second, all gray matter and white matter images from the DARTEL-imported space were used to create group templates using iterative algorithms and the deformation fields from each subject’s native space into the Montreal Neurological Institute (MNI) template. Third, the deformation fields were applied to the corresponding gray matter images to output the GMV images in the standard MNI space for all of the subjects. The GMV images then have voxel-wise correspondence across subjects. Total GMV, total white matter volume (WMV), total cerebrospinal fluid (CSF) volume, and total intracranial volume (TIV) for each subject were calculated using the SPM12 Util tool “Tissue Volumes” from the segmentation results.

### Statistical analysis

The general linear models (GLMs) were employed to compare total GMVs, total WMVs, total CSF volumes, and TIVs from all of the subjects with the independent variables of NC, MCI, AD groups, and confounding variables of age and gender.

The GLM was also used to determine the voxels in GMV maps with significant differences between 58 NC, 50 MCI and 40 AD subjects (Time 2) using the Statistical Nonparametric Mapping (SnPM13) toolbox [35] within SPM12. The SnPM approach was adopted in the image statistical analyses because of the potentially increased family-wise error (FWE) rates from the SPM cluster-level analyses [36, 37]. The nonparametric approach was demonstrated to be robust using a FWE rate of 5% [37]. In the GLMs, the group index (MCI or AD) was considered as the covariate of interest and the age, gender, and TIV were considered as confounding variables (covariates of no interest). One thousand random permutations were performed. The cluster-forming threshold was defined using a voxel-wise threshold of p < 0.001. The largest supra-threshold cluster sizes from all 1000 permutations were used to calculate the empirical distribution in order to correct for multiple comparisons among voxels. Only the significant clusters with FWEs of 5% were reported.

Automatic anatomical labeling (AAL) for SPM12 was used for the anatomical labeling of each cluster [38]. The intersection of each cluster and the anatomical region of interest (aROI) was calculated as two percentages: the percentage of cluster (%Cluster) and the percentage of ROI (%Region). %Cluster was calculated as the ratio of the number of voxels in the intersection to that in the cluster. %Region was calculated as the ratio of the number of voxels in the intersection to that in the aROI.

### Region of interest (ROI) derived from GMV changes

Clusters that showed significant GMV differences between NC and AD groups in the voxel-level analyses were considered as the atrophy ROI. Both left and right hippocampal ROIs were added as exploratory GMV ROIs because of frequently reported GMV loss in the hippocampus [13-15]. Because GMV atrophy was found in the left side from this study, the right hippocampal ROI was explored only if the left hippocampal ROI was not shown significant. The regional GMV for each ROI was calculated as the mean GMV over the region’s voxels.

### ROI derived from perfusion changes

A perfusion ROI was derived using the voxel-wise analysis with the voxels in CBF maps with significant differences between 58 NC, 50 MCI and 40 AD subjects (Time 2) using SPM12 from our prior cross-sectional study [21]. Specifically, we extracted the temporoparietal region as the perfusion ROI. Its precise location and the method deriving it can be found in the Supplementary Material (Supplementary Fig. 1). The temporoparietal region was also chosen because it was consistently reported to have an early perfusion/metabolism decrease in AD across different imaging modalities: ASL perfusion MRI, SPECT, and PET glucose metabolism. Because GMV atrophy was found in the left side from this study, only the left temporoparietal region was used.

### Potential confounding factors of perfusion in the high-risk AD cohort

AD patients with high genetic risk, i.e., those with the apolipoprotein E e4 (APOE4) allele in NC and MCI groups, have been shown with hyper-perfusion in the medial temporal lobes [39]. We therefore characterized the effect of APOE4 allele on brain perfusion in our high-risk AD cohort. For the 58 NC and 50 MCI participants at Time 2, 20 participants (12 NC, 8 MCI) carried the APOE4 allele (APOE+), including both homozygotes and heterozygotes; 73 participants (39 NC, 34 MCI) did not carry APOE4 allele (APOE-); 15 participants (7 NC, 8 MCI) did not have APOE tests. We compared perfusion maps and regional perfusion values in the left and right hippocampus regions between 20 APOE+ participants and 73 APOE- participants with age and gender as confounders. Additional comparisons were performed in the region-level analyses of hippocampus regions because the literature has reported hyper-perfusion in the hippocampus regions of APOE4 allele carriers [39] and regional analyses have increased signal-to-noise ratio (SNR) to detect the potential confounding factors. No significant perfusion difference was observed in the APOE4 carriers, indicating that the perfusion estimates in the high-risk AD cohort were not confounded by their APOE4 status.

### Relationship between GMVs (at Time 1 or Time 2) and longitudinal perfusion changes (from Time 2 to Time 3)

Multiple linear regression models were created to investigate whether the regional GMVs (Time 2) were related to longitudinal regional perfusion changes (from Time 2 to Time 3). Longitudinal perfusion changes from the 63 subjects in each ROI were the dependent variables. GMV values from the 63 subjects in either ROI at Time 2 were the independent variables. Age, gender, and time gap between two perfusion scans were considered as covariates. Multiple linear regression models were also created for the subjects with AD progression (i.e., not stable NC) to investigate whether the relationship was sensitive to AD progression. We also applied the above two multiple linear regression models to investigate whether the regional GMVs at Time 1 in 1997-1999 were related to longitudinal regional perfusion changes (from Time 2 to Time 3) after 2002. Forty subjects were used in these additional analyses because only 40 out of the 63 subjects received structural MRIs in 1997-1999. Given the disproportionate AD prevalence between genders [40], the interaction of gender and independent variable (regional GMVs at Time 2 in this case) was assessed for all investigated associations.

### Relationship between perfusion at Time 2 and longitudinal GMV changes (from Time 2 to Time 3)

Multiple linear regression models were created to investigate whether the regional perfusion values at Time 2 were related to longitudinal regional GMV changes (from Time 2 to Time 3). Longitudinal GMV changes from the 63 subjects in each ROI were the dependent variables. Regional perfusion values from the 63 subjects in either ROI at time 2 were the independent variables. Age, gender, and time gap between two structural MRI scans were considered as covariates. Multiple linear regression models were also performed for the subjects with AD progression (excluding those in the stable NC group). We cannot investigate whether the regional GM perfusion values at Time 1 in 1997-1999 are related to longitudinal regional GMV changes (from Time 2 to Time 3) after 2002 because no perfusion MRIs were acquired in 1997-1999.

### Relationship between longitudinal perfusion changes and longitudinal GMV changes (from Time 2 to Time 3)

Multiple linear regression models were created to investigate whether the longitudinal perfusion changes were related to longitudinal regional GMV changes from Time 2 to Time 3. Longitudinal perfusion changes from the 63 subjects in each ROI were the dependent variables. Longitudinal GMV changes from the 63 subjects in either ROI were the independent variables. Age, gender, and time gap between two structural MRI scans were considered as covariates.

**Table 1:** Demographic and cognitive scores at three time points.

	Scans at Time 1 (1997-1999)	No scans at Time 1 (1997-1999)	Scans at Time 2 (2002-2003)			Scans at Time 3 (2003-2009)
	n = 40 37 NC/3 MCI	n = 23 20 NC/3 MCI	n = 40 23 NC /11 MCI/6 AD	n = 63 29 NC/25 MCI /9 AD	n = 40 11 NC /18 MCI/11AD	n = 63 15 NC/30 MCI/18AD
**Age (years)**	78.6±3.8	78.3±4.0	84.0±3.9	83.8±3.8	86.2±3.9	86.3±3.8
**Gender (F, %)**	26 (65%)	12 (52.2%)	26 (65%)	38 (60.3%)	26 (65%)	38 (60.3%)
**Education (years)**	14.4±3.0	14.4±3.9	14.4±3.0	14.4±3.3	14.4±3.0	14.4±3.3
**Hypertension (%)**	18 (40%)	7 (30.4%)	18 (40%)	25 (39.7%)	18 (40%)	25 (39.7%)
**Diabetes (%)**	6 (15%)	0 (0%)	6 (15%)	6 (9.5%)	6 (15%)	6 (9.5%)
**Heart disease (%)**	8 (20%)	5 (21.7%)	8 (20%)	13 (20.6%)	8 (20%)	13 (20.6%)
**3MSE scores**	96.3±4.1	96.2±3.5	93.1±6.0	93.4±5.7	91.4± 8.0	91.8±7.3
**Follow-up time**	N/A	N/A	5.4±0.6	N/A	2.3±1.6	2.3±1.7

## RESULTS

The demographic and cognitive information of the 148 subjects at Time 2 that were used to generate the GMV ROIs for abnormal GM atrophy in AD are listed in Supplementary Table 1 and reported in our previous publication [21]. No significant differences were observed for age, gender, and education between the three cognitive groups. Subjects with AD had lower 3MSE scores compared to NC and MCI subjects (p < 0.0001).

The Table 1 summarizes the demographic and general cognitive information of the 63 subjects at two time points and 40 out of the 63 subjects at three time points. The 40 subjects had an average time gap of 5.4±0.6 years from Time 1 to Time 2 and of 2.3±1.6 years from Time 2 to Time 3. The 63 subjects had an average follow-up time of 2.3±1.7 years from Time 2 to Time 3. No significant differences were observed for age, gender, education, hypertension, diabetes, and heart disease between 40 subjects with Time 1 data and 23 subjects without Time 1 data.

### Comparison of global GMVs at Time 2

We found an association of age with the four total brain volume measures (β= -0.42% per year, p = 0.0011 for the total GMV;β= -0.34% per year, p = 0.0042 for the total WMV;β= +0.31% per year, p = 0.042 for the total CSF volume;β= -0.46% per year, p = 0.079 marginal for the TIV, respectively) and of gender (smaller in females) with the above four total brain volume measures (β= -0.040 liters, p < 0.0001;β= -0.061, p < 0.0001;β= -0.078 liters, p < 0.0001;β= -0.18 liters, p < 0.0001, respectively). However, no significant differences of total GMVs, total WMVs, total CSF volumes, and TIVs were observed between NC and MCI, between NC and AD, and between MCI and AD.

**Figure 2.**
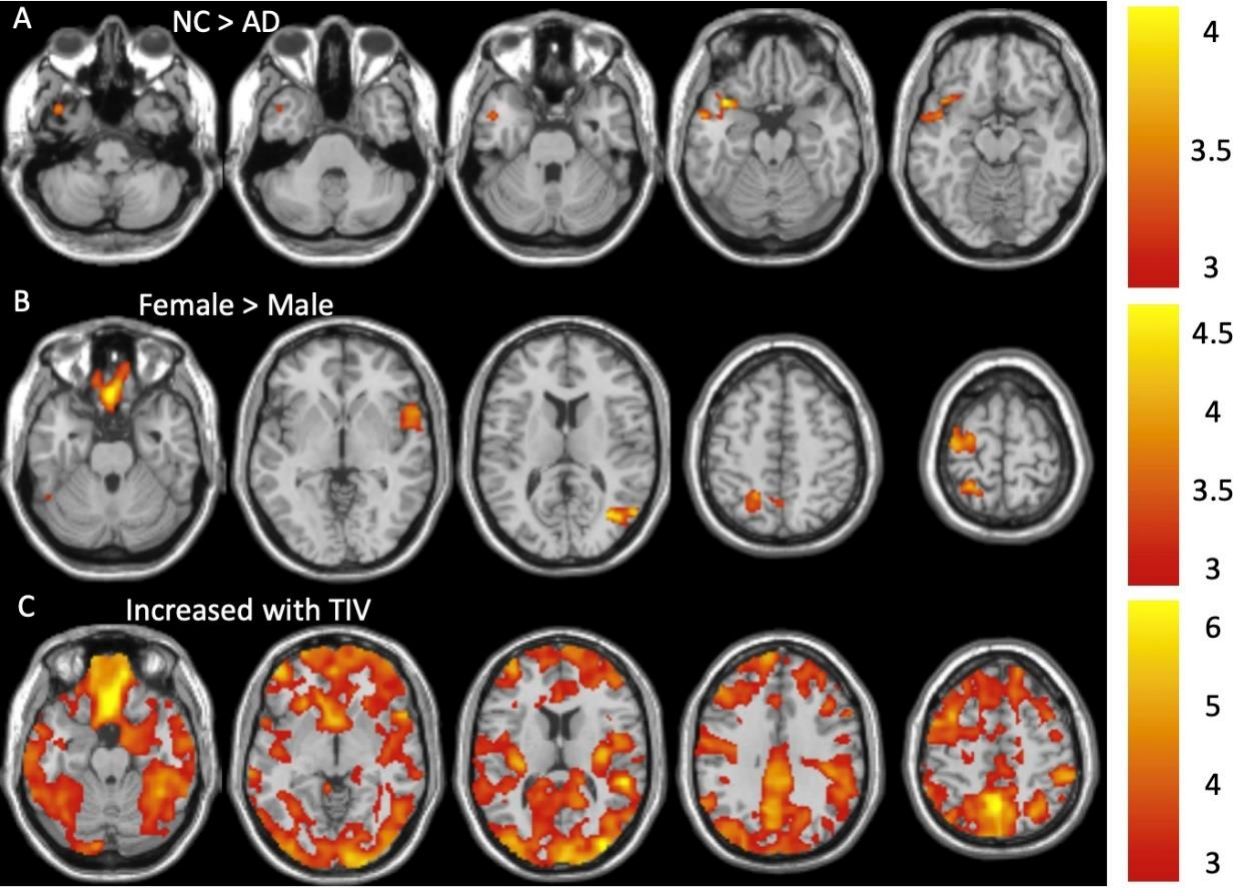
**The AD group had significant GMV decreases (A) in the temporal pole cluster (Cluster 1) compared with the NC group after adjusting for age, gender, and TIV effects**. Females showed significantly larger GMVs (B) in the rectus (Cluster 2), fusiform (Cluster 3), rolandic operculum and inferior frontal (Cluster 4), middle temporal (Cluster 5), precuneus and superior parietal (Cluster 6), and precentral (Cluster 7) regions after adjusting for age and TIV effects. Larger GMVs were associated with larger TIVs (C) in almost the entire brain (Cluster 8) after adjusting for age and gender effects. The color bars show the range of t-values.

### Comparison of GMV maps at Time 2

The AD subjects had decreased GMVs compared to NCs in the temporal pole region (Fig. 2A). No significant differences in GMVs were observed between the AD and MCI groups and between NC and MCI groups. In females, we also observed significantly larger GMVs in the rectus, fusiform, rolandic operculum, middle temporal, superior parietal, precuneus, and predental regions (Fig. 2B) after adjusting for TIV and age. Almost all of the local GMVs were positively correlated with TIVs (Fig. 2C). Cluster statistics of these clusters are shown in Table 2. However, the temporal pole cluster with decreased GMVs in AD (1178 voxels) is small. To increase the SNR for further regional analyses, we derived a larger cluster (3865 voxels) with decreased GMVs in AD by using a similar SnPM analysis with a more relaxed voxel-level p-value threshold of 0.005. The cluster-level FWE-corrected p-value threshold remained as 0.05. The cluster remained in the temporal pole region, as shown in Supplementary Figure 2, in which its cluster statistics is also reported.

### Correlation of GMVs (at Time 2) with longitudinal perfusion decline (from Time 2 to Time 3)

**Figure 3.**
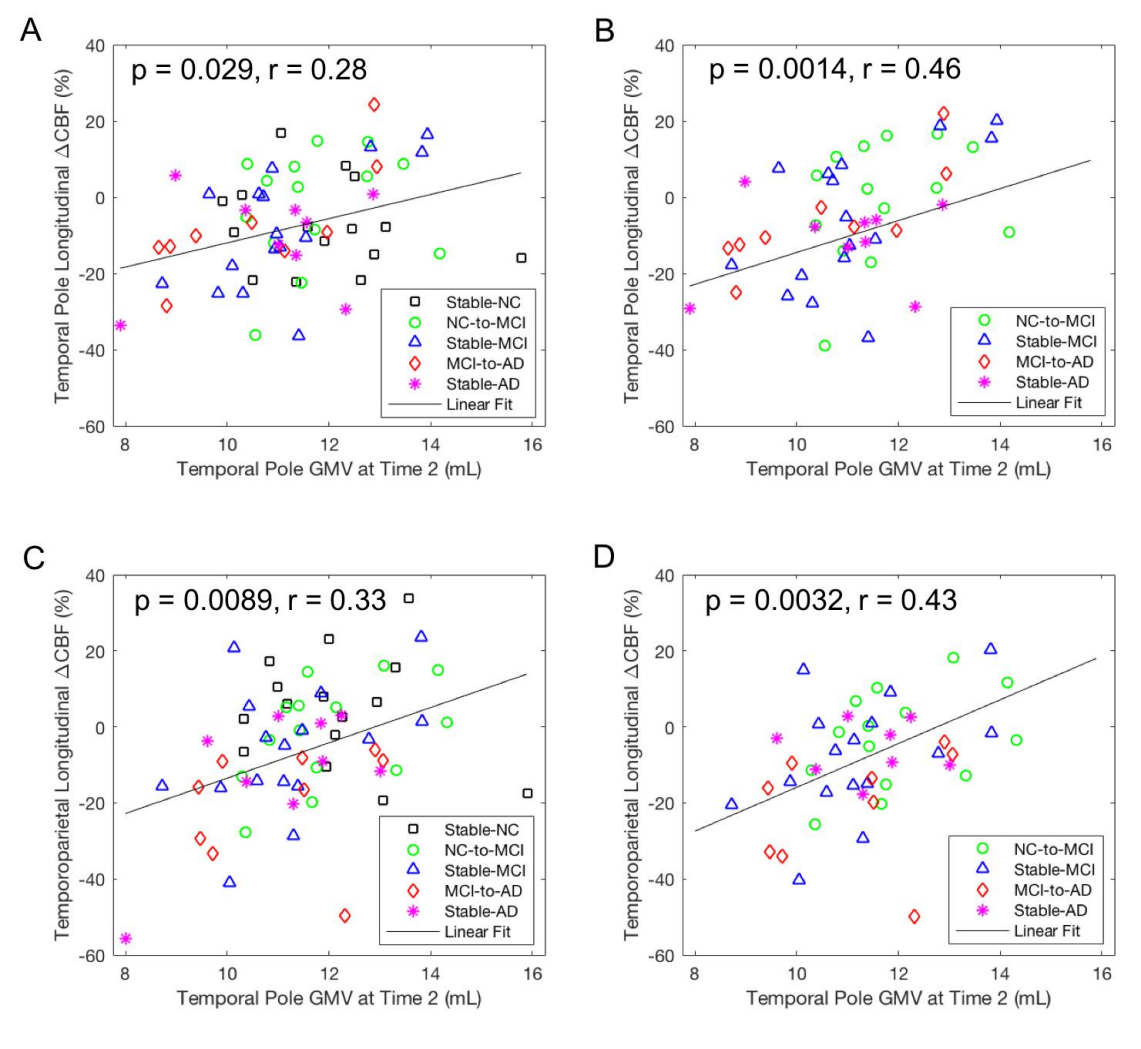
**Correlation of longitudinal CBF changes from Time 2 to Time 3 in the temporoparietal region with GMV values at Time 2 after adjusting for age, gender, and time gap from Time 2 to Time 3**. Longitudinal CBF changes in the temporoparietal region were significantly associated with (A) temporoparietal GMV values for all of the subjects (n = 63), (B) temporoparietal GMV values for the subjects with AD progression (n = 48), (C) temporal pole GMV values for all of the subjects (n = 63), and (D) temporal pole GMV values for the subjects with AD progression (n = 48).

For all of the subjects (n = 63), GMVs in the temporal pole region at Time 2 were significantly associated with longitudinal perfusion declines from Time 2 to Time 3 in this region (Fig. 3A, p = 0.029, r = 0.28) and in the temporoparietal region (Fig. 3C, p = 0.0089, r = 0.33), and the association remained significant only in the temporoparietal region after FWE correction. For the subjects with AD progression (i.e., not stable NC, n = 48), GMVs in the temporal pole region at Time 2 were significantly associated with longitudinal perfusion declines from Time 2 to Time 3 in this region (Fig. 3B, p = 0.0014, r = 0.46) and in the temporoparietal region (Fig. 3D, p = 0.0032, r = 0.43), and both associations remained significant after FWE correction.

For our exploratory aim, left hippocampal GMVs at Time 2 were significantly associated with longitudinal perfusion declines in the temporoparietal region from Time 2 to Time 3 for all of the subjects (Supplementary Fig. 3A, p = 0.027, r = 0.29) and for the subjects with AD progression (Supplementary Fig. 3B, p = 0.012, r = 0.37). However, after FWE correction, this association remained significant only for the subjects with AD progression. More interestingly, the slopes for the subjects with AD progressions were larger than those derived using all of the subjects. However, the differences between the two slopes were not statistically significant.

For all of the subjects (n = 63), significant interaction between gender and temporal pole GMV at Time 2 (b = 2.76, p = 0.0052) and marginally significant interaction between gender and hippocampal GMV at Time 2 (b = 2.18, p = 0.088) were observed for longitudinal perfusion declines in the temporoparietal region. The interaction between gender and temporal pole GMV remained significant after FWE correction. Specifically, the association between the temporal pole GMV at Time 2 and temporoparietal CBF changes from Time 2 to Time 3 was significant in females (b = 2.17, p = 0.0013) but not in males (b = -0.22, p = 0.78). Marginally significant interaction between gender and hippocampal GMV at Time 2 (b = 2.18, p = 0.088) were observed for longitudinal perfusion declines in the temporoparietal region. The association between the hippocampal GMV and temporoparietal CBF changes was significant in females (b = 1.93, p = 0.030) but not in males (b = 0.78, p = 0.50).

**Table 2:** Clusters with significantly decreased GMVs in the AD group relative to the NC group (Cluster 1) after adjusting for TIV, age, and gender; larger GMVs in females than males (Clusters 2-7) after adjusting for TIV and age; larger GMVs associated with larger TIVs (Clusters 8) after adjusting for age and gender.

	Cluster	N voxels	Peak-t	Peak-t MNI coordinates	Anatomical locations	%Cluster	%Region
**1**	Temporal pole [L] (NC>AD)	1178	4.08	-38, 12, -20	Temporal Lobe Temporal_Pole_Sup_L Temporal_Mid_L Temporal_Sup_L Temporal_Inf_L Temporal_Pole_Mid_L Insula Insula_L Frontal Lobe Frontal_Inf_Orb_L	29.56 21.57 8.52 8.26 3.20 11.58 3.99	17.28 3.28 2.79 1.94 3.18 4.68 1.78
**2**	Female > Male	1905	4.82	-2, 27, -28	Frontal Lobe Rectus_R Rectus_L Frontal_Sup_Orb_R Frontal_Sup_Orb_L lfactory_R	24.54 29.61 6.88 6.69 0.37	35.44 24.78 7.42 7.48 1.38
**3**		1097	4.23	-36,-51, -15	Occipital Lobe Fusiform_L Occipital_Inf_L Temporal Lobe Temporal_Inf_L Cerebelum Cerebelum_6_L	76.75 6.83 12.31 3.80	21.86 4.78 2.53 1.47
**4**		1007	4.01	56, 12, 3	Frontal Lobe Frontal_Inf_Oper_R Rolandic_Oper_R Frontal_Inf_Tri_R Temporal Lobe Temporal_Pole_Sup_R Temporal_Sup_R Insula Insula_R	36.67 22.00 1.61 14.67 7.69 11.27	14.65 9.24 0.42 6.13 1.37 3.56
**5**		1189	4.75	34, -69, 15	Temporal Lobe Temporal_Mid_R Occipital Lobe Occipital_Mid_R Occipital_Sup_R Cuneus_R Parietal Lobe Precuneus_R	40.51 17.63 5.11 2.56 3.50	6.83 6.24 2.69 1.33 0.80
**6**		1498	4.12	-26,-48, 66	Parietal Lobe Parietal_Sup_L Precuneus_L Precuneus_R Postcentral_L Parietal_Inf_L Occipital Lobe Cuneus_R	54.15 28.56 4.01 2.96 1.91 1.24	27.46 8.47 1.29 0.80 0.82 0.91
**7**		1357	4.10	-38,-15, 66	Frontal Lobe Precentral_L Frontal_Sup_L	80.03 9.16	17.83 2.00
**8**	Increased with TIV	226769	7.06	-2, 27, -27	Frontal Lobe Frontal_Mid_L Frontal_Mid_R Frontal_Sup_R Frontal_Sup_L Precentral_L Frontal_Sup_Medial_L Frontal_Sup_Medial_R Supp_Motor_Area Frontal_Inf_Orb_L Frontal_Inf_Orb_R Frontal_Mid_Orb_R Rolandic_Oper_R Rectus_L Frontal_Sup_Orb_L Frontal_Med_Orb_R Frontal_Inf_Tri_R Frontal_Mid_Orb_L Supp_Motor_Area_L Frontal_Inf_Tri_L Rectus_R Frontal_Med_Orb_L Frontal_Sup_Orb_R Rolandic_Oper_L Frontal_Inf_Oper_R Precentral_R Olfactory_R Olfactory_L Frontal_Inf_Oper_L Temporal Lobe Temporal_Mid_R Temporal_Inf_R Temporal_Mid_L Temporal_Inf_L Temporal_Sup_L Temporal_Sup_R Temporal_Pole_Sup_R Temporal_Pole_Mid_R Temporal_Pole_Sup_L Amygdala_R Heschl_L Temporal_Pole_Mid_L Heschl_R Occipital Lobe Occipital_Mid_L Fusiform_R Calcarine_L Fusiform_L Occipital_Mid_R Calcarine_R Cuneus_L Lingual_R Cuneus_R Occipital_Sup_L Occipital_Sup_R Occipital_Inf_R Lingual_L Occipital_Inf_L Parietal Lobe Precuneus_R Precuneus_L Parietal_Sup_L SupraMarginal_R Angular_R Postcentral_L Parietal_Inf_L Parietal_Sup_R Parietal_Inf_R Angular_L Postcentral_R Paracentral_Lobule_R SupraMarginal_L Paracentral_Lobule_L Insula Insula_R Insula_L Cerebelum Cerebelum_Crus1_R Cerebelum_8_R Cerebelum_6_R Cerebelum_Crus2_R Cerebelum_6_L Cerebelum_Crus1_L Cerebelum_9_L Cerebelum_7b_R Cerebelum_4_5_L Cerebelum_8_L Cerebelum_4_5_R Cerebelum_9_R Cerebelum_Crus2_L Vermis_4_5 Vermis_6 Cerebelum_7b_L Limbic Lobe Cingulum_Mid_L Cingulum_Mid_R Cingulum_Ant_L ParaHippocampal_R Cingulum_Ant_R Cingulum_Post_L Hippocampus_R Cingulum_Post_R Hippocampus_L ParaHippocampal_L Basal Ganglia Thalamus_R Caudate_L Putamen_R Caudate_R Putamen_L Pallidum_L Pallidum_R	2.82 2.55 2.21 1.70 1.70 1.59 1.14 1.14 1.00 0.93 0.76 0.75 0.73 0.71 0.71 0.69 0.68 0.68 0.66 0.64 0.61 0.60 0.47 0.44 0.39 0.24 0.23 0.15 2.56 2.46 2.01 1.70 1.24 1.20 0.63 0.50 0.26 0.21 0.18 0.15 0.14 2.36 1.77 1.45 1.37 1.35 1.03 1.01 1.00 0.98 0.91 0.87 0.75 0.73 0.69 2.24 2.09 1.11 1.07 1.05 0.97 0.96 0.93 0.65 0.55 0.47 0.37 0.31 0.04 1.23 0.95 1.05 0.94 0.69 0.54 0.44 0.39 0.24 0.15 0.12 0.10 0.10 0.02 0.01 0.01 0.01 0.01 1.01 0.91 0.60 0.53 0.52 0.38 0.33 0.27 0.07 0.02 0.34 0.24 0.18 0.15 0.09 0.02 0.01	67.65 58.37 63.44 55.40 56.26 62.13 62.56 56.13 69.17 64.08 87.49 66.49 100.00 86.91 96.96 37.47 89.52 36.75 30.72 99.86 98.75 70.71 56.26 37.31 13.49 98.27 97.50 17.05 67.61 80.52 47.47 62.09 63.02 44.69 55.15 49.36 23.73 99.19 94.22 23.31 65.46 84.25 82.24 75.24 69.52 75.21 64.53 77.71 50.86 80.89 78.33 72.18 89.28 41.09 85.97 79.88 68.99 62.95 63.22 70.37 29.08 46.13 49.32 56.65 54.99 14.54 52.39 28.42 3.11 81.41 59.84 46.49 47.57 44.85 29.57 30.46 17.55 32.80 32.96 12.62 6.46 13.70 2.97 1.16 1.80 3.23 1.71 60.99 48.66 50.00 55.03 45.85 94.82 40.70 94.93 8.47 2.45 37.18 29.00 20.21 17.91 10.40 9.21 2.14

### Correlation of GMVs (at Time 1) with longitudinal perfusion decline from average 5.4 years later

**Figure 4.**
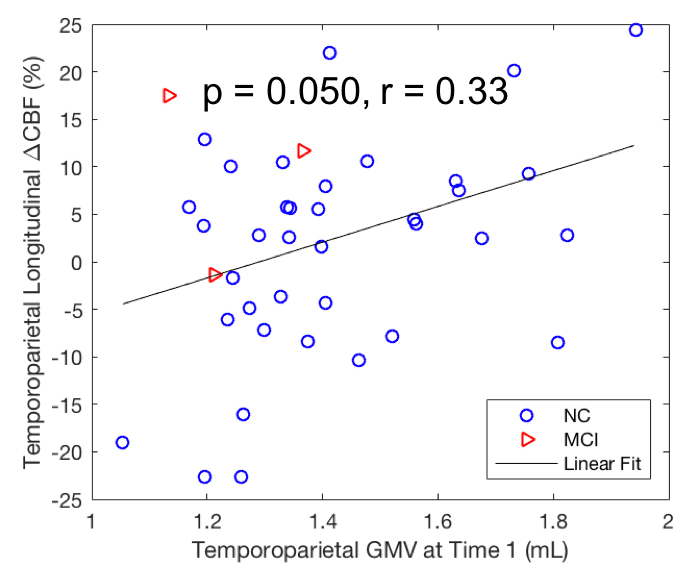
**Correlation of longitudinal CBF changes from Time 2 to Time 3 with GMV values at Time 1 after adjusting for age, gender, and time gap**. Longitudinal CBF changes in the temporoparietal region were significantly correlated with its own GMV. Time 2 was on average 5.4 years later than Time 1 (n = 40).

For all of the 40 subjects, GMVs in the left temporoparietal region at Time 1 were significantly associated with its longitudinal perfusion declines from Time 2 to Time 3 (Fig. 4, p = 0.050, r = 0.33). However, after FWE correction, this association at Time 1 was no longer significant. GMVs in either the left hippocampus or the right hippocampus region were not associated with longitudinal perfusion declines from Time 2 to Time 3 in its region and in the temporoparietal region (p > 0.05). No significant interaction was observed between gender and left hippocampal GMV at Time 1 (p = 0.31) for all of the subjects.

### Correlation of perfusion values at Time 2 with longitudinal GMV changes (from Time 2 to Time 3)

**Figure 5.**
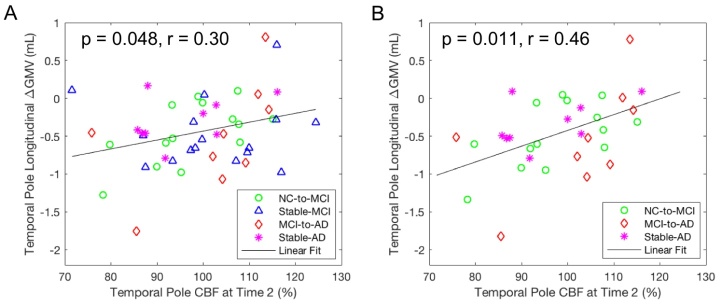
**Correlation of longitudinal GMV changes from Time 2 to Time 3 with CBF values at Time 2 after adjusting for age, gender, and time gap**. Longitudinal GMV changes in the temporal pole region were significantly correlated with its CBF values for (A) the subjects with AD progression (n = 48) and (B) the subjects with definite AD progression (n = 32).

**Figure 6.**
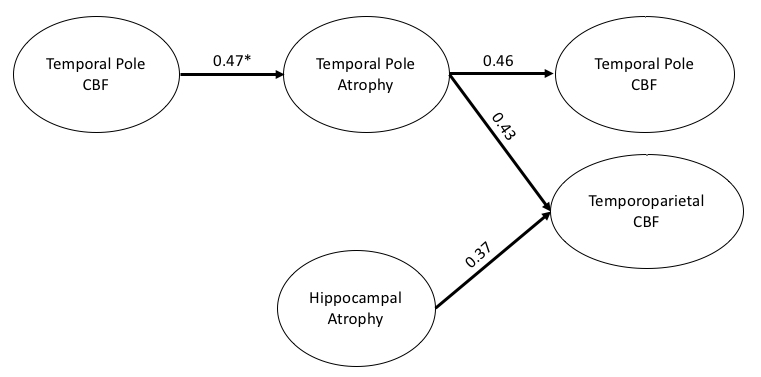
**Correlation model of relationship among temporal pole CBF, temporoparietal CBF, temporal pole atrophy, and hippocampal atrophy**. Path weights are partial correlation coefficients after adjusting for age, gender, and time gap for all of the subjects with AD progression (n = 48, excluding the stable NC subjects). The direction of arrows is from an early event to a later event. * Stands for partial correlation coefficients for all of the subjects with definite AD progression (n = 32, excluding the stable NC and stable MCI subjects).

Perfusion values at Time 2 in the temporal pole region were marginally associated with its own longitudinal GMV changes (from Time 2 to Time 3) for all of the subjects (p = 0.079, r = 0.23) but significantly associated for the subjects with AD progression (Fig. 5A, p = 0.048, r = 0.30). Considering that there was not much cognitive progression in the stable-MCI group, we excluded the stable-MCI subjects to explore this association for the subjects with definite AD progression. After excluding the stable-MCI subjects, we found a significant association of perfusion values at Time 2 in the temporal pole region with its own longitudinal GMV changes for the subjects with definite AD progression (Fig. 5B, p = 0.011, r = 0.47) and the association remained significant after FWE correction. Perfusion values in either hippocampus at Time 2 were not associated with longitudinal GMV changes (from Time 2 to Time 3) (p > 0.05). No significant interaction was observed between gender and temporal pole perfusion values at Time 2 (p = 0.98) for all of the subjects. Fig. 6 summarizes the correlation model derived among temporal pole CBF, temporoparietal CBF, temporal pole atrophy, and hippocampal atrophy.

### Correlation of longitudinal perfusion changes with longitudinal GMV changes (from Time 2 to Time 3)

Longitudinal CBF changes in the temporal pole and left hippocampus were negatively associated with longitudinal changes of GMV values in the same regions (Supplementary Fig. 4A, p = 0.018, r = -0.30 for the temporal pole; Supplementary Fig. 4B, p = 0.027, r = -0.29 for the left hippocampus). However, after FWE correction, these associations lacked significance.

## DISCUSSION

Our results showed that AD was associated with marked atrophy in the left temporal pole region. The atrophy in the temporal pole is consistent with the location of neuronal loss and neurofibrillary tangles in AD [41], the distribution of temporal lobe atrophy with advancing disease [42], and the progression of TDP-43 pathology in AD: stage 2 for atrophy in hippocampus and/or entorhinal cortex and stage 3 for extension to the anterior temporal pole cortex [43]. The left anterior temporal pole has also been associated with impairment of semantic memory in AD [44]. However, the observed temporal pole region finding is different from frequently reported early AD atrophic regions: hippocampal and entorhinal areas [45-48].

By contrast, using the 1997-1999 CHS-CS cohort, we observed significant hippocampal atrophy in AD in a prior publication [49]. It is worth noting that the current study used the 2002-2003 CHS-CS cohort data to derive the AD atrophic regions and therefore the current study has subjects 4 to 5 year older than our prior study that used 1997-1999 data [49]. In addition, our prior 1997-1999 cohort used NCs who remained cognitively normal for at least 5 years after their MRI scans, while NCs from this cohort were considered as cognitively normal subjects at the time of their MRI scans. Therefore, there may not be a sharp boundary between NC and MCI groups as corroborated by no difference in the GMV between these two groups. We postulated that the observed atrophy in the temporal pole region reflects brain structural atrophy in a more advanced stage of AD compared to our prior 1997-1999 cohort.

We found that atrophy in the left temporal pole and left hippocampal regions at Time 2 was correlated with subsequent perfusion decline in the temporoparietal region. Our correlation results with longitudinal perfusion changes extend the prior cross-sectional study with an association between GMVs in the medial temporal cortex and perfusion values in the combined AD hypoperfusion area [20]. Despite no statistical significance, we observed a faster perfusion decline rate when the stable normal subjects were excluded. We also observed that atrophy in the left temporal pole at Time 2 was correlated with its own subsequent perfusion decline. Our results suggest that GMV in the temporal lobe is a strong indicator of future perfusion declines in the local and adjacent vessel territory. A faster rate of perfusion decline may occur as AD progresses. Brain atrophy in the temporal lobe can cause reduced demand for perfusion from the posterior cerebral artery (PCA). The temporoparietal region, located in the border zone of the PCA and middle cerebral artery (MCA), is very vulnerable to conditions such as small vessel diseases and impaired vessel autoregulation. Brain atrophy was shown to be predicted from the tau pathology [50]. These data support that the atrophy (and maybe tau) in the temporal lobe may be a major driver of hypoperfusion and highlight the potential relevance of temporal lobe atrophy to predict the progression of hypoperfusion in future clinical trials. Nevertheless, the negative association between GMV changes and CBF changes in the temporal pole and hippocampus regions (i.e., the larger GMV values declined, the higher CBF values increased) indicates strong perfusion compensation to save neuronal degeneration at the early stage of AD progression. The perfusion compensatory mechanism is supported by tau-induced abnormal angiogenesis with increased blood vessel density in animal models [51].

We also investigated the potential cause of atrophy in the temporal lobe. Longitudinal GMV changes from Time 2 to Time 3 were found to be associated with brain perfusion in the temporal pole region but not in the hippocampal region at Time 2. Failure to find the association between hippocampal perfusion and its atrophy may be caused by the cohort that we analyzed being in a relatively advanced stage and relative older age group as mentioned earlier. However, the initial hypoperfusion appearing in the hippocampus and entorhinal regions was supported by animal models. Capillary degeneration was shown to develop experimentally in the CA1 hippocampal sector and entorhinal cortex of rat brain and were associated with spatial memory deficits when chronic cerebral hypoperfusion was induced in aging animals for a year [52, 53]. Our findings support the critically attained threshold of cerebral hypoperfusion (CATCH) hypothesis of Alzheimer’s pathogenesis [54]: advanced aging in the presence of a vascular risk factor can create a CATCH that disturbs regional (temporal pole) brain microcirculation and impairs optimal energy metabolism to maintain healthy brain cell function.

In females, brain atrophy in the temporal pole and hippocampal regions was found to be associated with a faster perfusion decline in the temporoparietal region. A perfusion decline was an early event that heralds the decline of cognitive function in AD [8]. Therefore, our finding that females are more susceptible to the perfusion decline directly contributes to the understanding for the disproportionately higher prevalence of females with AD than males [40]. However, the increased prevalence of AD among women likely arises through a combination of factors, including sex hormones [55], brain structure [56], neuroinflammation [57], APOE genes [58], and life experiences [59]. More research is required to explain the increased risks of women in developing AD and tailor AD treatments to women.

Our results identified temporal dynamics between the decline of cerebral blood flow and brain atrophy (neurodegeneration) during AD progression, supporting the involvement of cerebrovascular pathology in AD. CBF reduction may emerge from reduced capillary densities [60, 61] and the prolonged vasoconstriction of brain blood vessels in AD [4, 62], which may be related to the leakage of neurotoxic products into the brain through a defective blood-brain barrier [63]. When CBF diminishes, damage to neurons (brain atrophy) occurs and brain cognitive functions corresponding to these neurons are compromised [64]. The temporoparietal and posterior cingulate cortex regions demonstrated both declined CBF and reduced glucose metabolism from the same AD cohort [9-12], suggesting the close link between CBF and glucose metabolism. Hypometabolism in the medial parietal and lateral temporoparietal regions is an AD biomarker of neurodegeneration [65]. Measurement of CBF in these regions with noninvasive means (e.g., ASL) may be a valuable option to investigate the vascular role in AD pathology.

This study has some limitations. First, the participants were classified into different groups based on adjudications of their cognitive status, not from biomarker-confirmed (i.e., A+/T+) diagnosis of AD [66]. Therefore, misclassification of AD patients may have occurred. Second, the CBF images were acquired using the 2D multi-slice CASL technique at 1.5T. The subsequent availability of PCASL, 3T MRI, and background suppressed 3D acquisitions have resulted in significant improvements in temporal SNR and potential sensitivity to CBF differences [67, 68]. Nevertheless, in this study, we were able to detect associations between atrophy and subsequent CBF decline, and between reduced CBF and subsequent atrophy using CASL. Third, history of structural central nervous system lesions, small vessel diseases, and stroke was excluded from the perfusion MRI study, despite individuals with a history of stroke having a 59% increased risk of developing AD [69]. Therefore, our findings cannot be extended to the broader population with vascular comorbidities. Further studies are needed to clarify whether vessel diseases and atrophy contribute independently to perfusion decline.

Our data suggested that hypoperfusion in the temporal pole region is associated with atrophy in this region and, subsequently, to perfusion declines in both the temporal pole region and temporoparietal region. We postulate that CBF reduction occurs as an early event, and the hippocampus and temporal lobe are generally more susceptible to hypoperfusion than other cortical regions [70]. However, we have not observed a direct association of hippocampal CBF and its atrophy because of missing CBF images in the Time 1 dataset. Future studies are warranted to study this crucial relationship.

In summary, hypoperfusion in the temporal pole preceding its atrophy suggests that hypoperfusion in the region may be an early event driving atrophy. Temporoparietal and temporal pole hypoperfusion follows atrophy in the temporal pole, indicating that the atrophy (and maybe tau) in the temporal pole may be a major driver of later hypoperfusion in the temporoparietal and temporal pole regions. The findings highlight the potential relevance of temporal lobe atrophy to predict the AD progression of hypoperfusion for future clinical trials.

## Supplementary Materials

The Supplementary data can be found online at: www.aginganddisease.org/EN/10.14336/AD.2023.0430.


