# Supplementary material for: Prospective Longitudinal Perfusion in Probable Alzheimer’s Disease Correlated with Atrophy in Temporal Lobe
Source: Aging Dis. 2024 Aug 1;15(4):1855–71. doi: 10.14336/AD.2023.0430 (PMC11272196; doi:10.14336/AD.2023.0430)
Supplement: Supplementary file 1 [file AD-15-4-1855-s.pdf]

SUPPLEMENTARY DATA

Prospective Longitudinal Perfusion in Probable Alzheimer's Disease Correlated with Atrophy in Temporal Lobe

Tony D. Zhou, Zongpai Zhang, Arvind Balachandrasekaran, Cyrus A. Raji, James T. Becker, Lewis H Kuller, Yulin Ge, Oscar L. Lopez, Weiying Dai, H. Michael Gach

© 2023. Zhou TD et al. Published online at http://www.aginganddisease.org/EN/10.14336/AD.2023.0430

SUPPLEMENTARY DATA

Supplemental Table 1. Demographic and cognitive scores at Time 2.

**Time 2 (n = 148)**			
	NC (n=58)	MCI (n=50)	AD (n=40)
Age (years)	83.4±3.7	84.5±3.6	84.1±3.5
Gender (F, %)	31 (53%)	32 (64%)	28 (70%)
Education (years)	14.6±2.8	14.3±3.4	13.3±2.9
Hypertension (%) †	25 (43%)	24 (49%)<br>(n'=49)	21 (53%)
Diabetes (%)	9 (16%)	7 (14%)	3 (8%)
Heart Disease (%)†	14 (24%)	11 (22%)	5 (13%)<br>(n'=39)
3MSE scores†	**95.0±3.9**<br>(n'=54)	93.3±6.2<br>(n'=44)	**83.6±10.0**<br>(n'=39)

† 3MSE scores, hypertension and heart disease cases were not completed for all the subjects. The actual number of subjects with valid records is indicated inside the brackets. Subjects with AD had lower 3MSE scores compared to NC subjects ($p < 0.0001$, shown in bold fonts).

© 2023. Zhou TD et al. Published online at http://www.aginganddisease.org/EN/10.14336/AD.2023.0430

SUPPLEMENTARY DATA

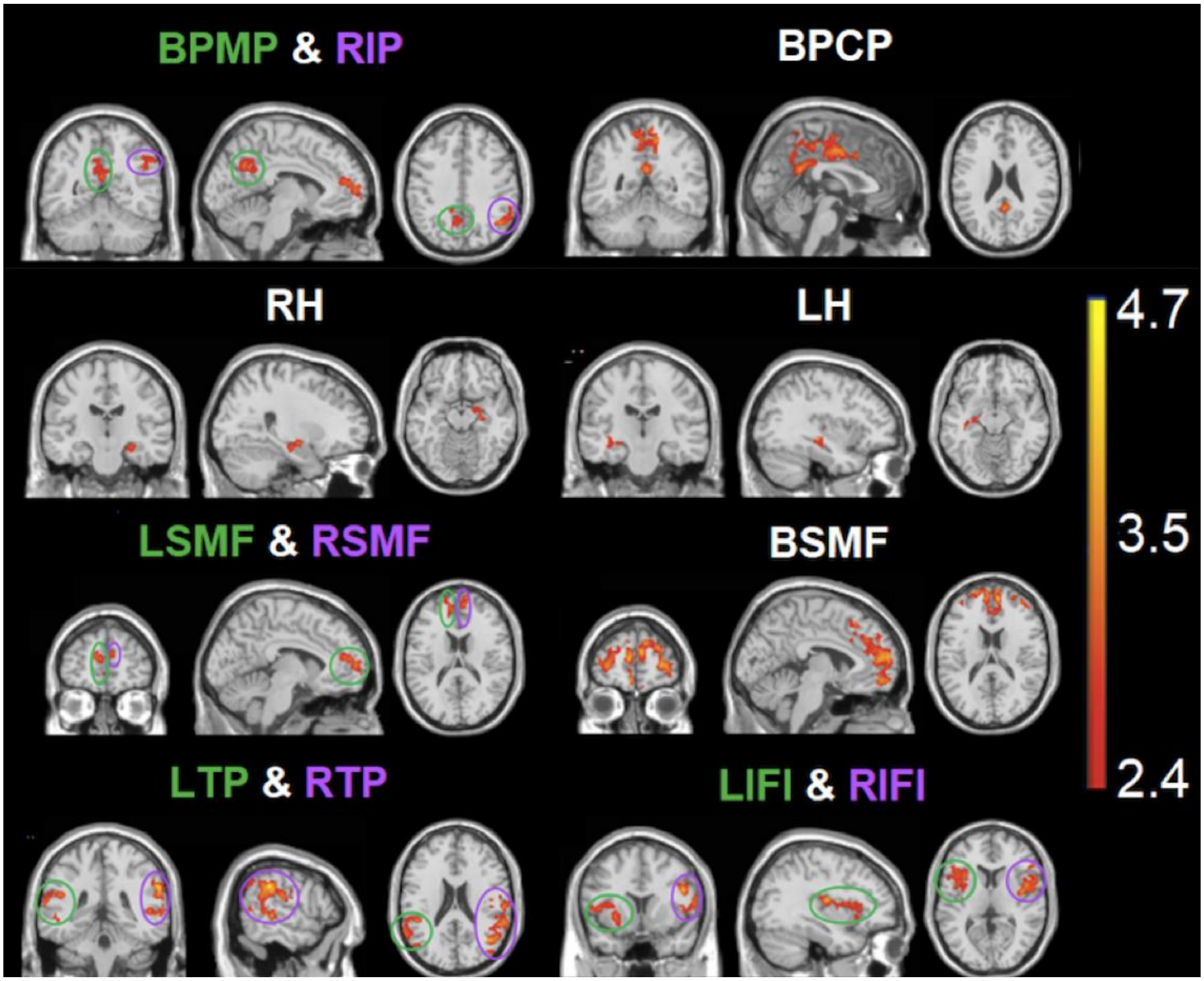

Supplementary Figure 1. Twelve regions of interest (ROIs) were identified from the voxel-by-voxel analysis at Time 2: right inferior parietal (RIP), bilateral posterior and middle cingulate and parietal regions (BPMP), bilateral posterior cingulate extending to the precuneus (BPCP), right hippocampus (RH), left hippocampus (LH), right superior medial frontal (RSMF), left superior medial frontal (LSMF), bilateral superior medial frontal (BSMF), right temporoparietal (RTP), left temporoparietal (LTP), right inferior frontal and insular (RIFI), left inferior frontal and insular (LIFI). LTP was used as the perfusion ROI in the study.

© 2023. Zhou TD et al. Published online at http://www.aginganddisease.org/EN/10.14336/AD.2023.0430

SUPPLEMENTARY DATA

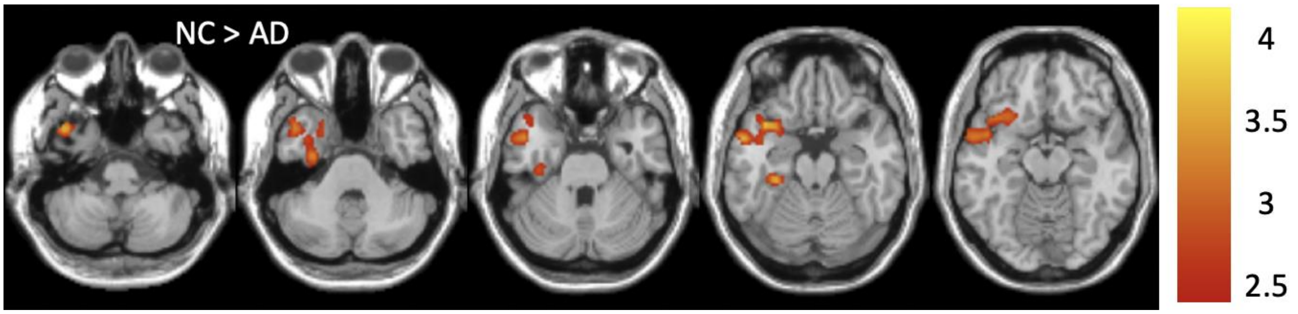

Cluster	N voxels	Peak-t	Peak-t MNI coordinates	Anatomical locations	%Cluster	%Region
Temporal pole [L]<br>(NC > AD)	3865	4.08	-38, 12, -20	**Temporal lobe**<br>Temporal_Pole_Sup_L<br>Temporal_Mid_L<br>Temporal_Pole_Mid_L<br>Temporal_Inf_L<br>**Occipital lobe**<br>Fusiform_L<br>**Insula**<br>Insula_L<br>**Limbic System**<br>ParaHippocampal_L<br>**Frontal lobe**<br>Frontal_Inf_Orb_L	 20.65<br>13.53<br>9.78<br>8.90<br><br>17.49<br><br>8.33<br><br>3.13<br><br>1.91	 26.20<br>4.46<br>21.12<br>4.54<br><br>12.35<br><br>7.31<br><br>5.22<br><br>5.51

Supplementary Figure 2. The AD group had significant GMV decreases in the temporal pole cluster (the top panel) compared with the NC group after adjusting for age, gender, and TIV effects using the SnPM analysis with a voxel-level p-value threshold of 0.005 and a cluster-level FWE-corrected p-value threshold of 0.05. The cluster statistics of the temporal pole cluster are shown in the bottom panel.

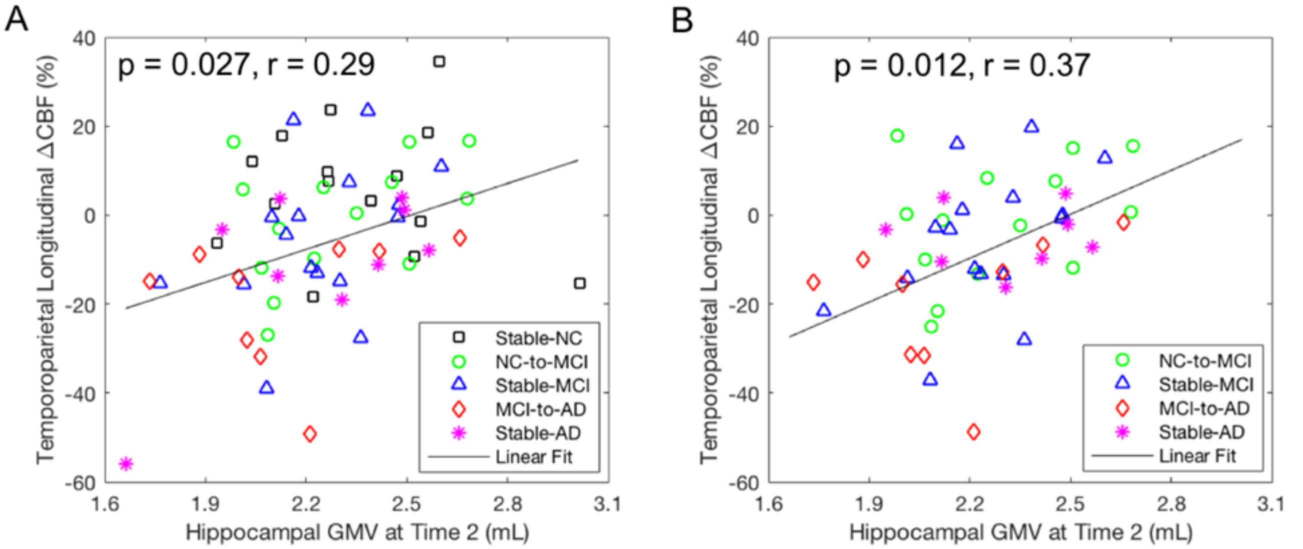

Supplementary Figure 3. Correlation of longitudinal CBF changes from Time 2 to Time 3 in the temporoparietal region with GMV values at Time 2 after adjusting for age, gender, and time gap. Longitudinal CBF changes in the temporoparietal region were significantly associated with (A) hippocampal GMV values for all of the subjects (n = 63), and (B) hippocampal GMV values for the subjects with AD progression (n = 48).

© 2023. Zhou TD et al. Published online at http://www.aginganddisease.org/EN/10.14336/AD.2023.0430

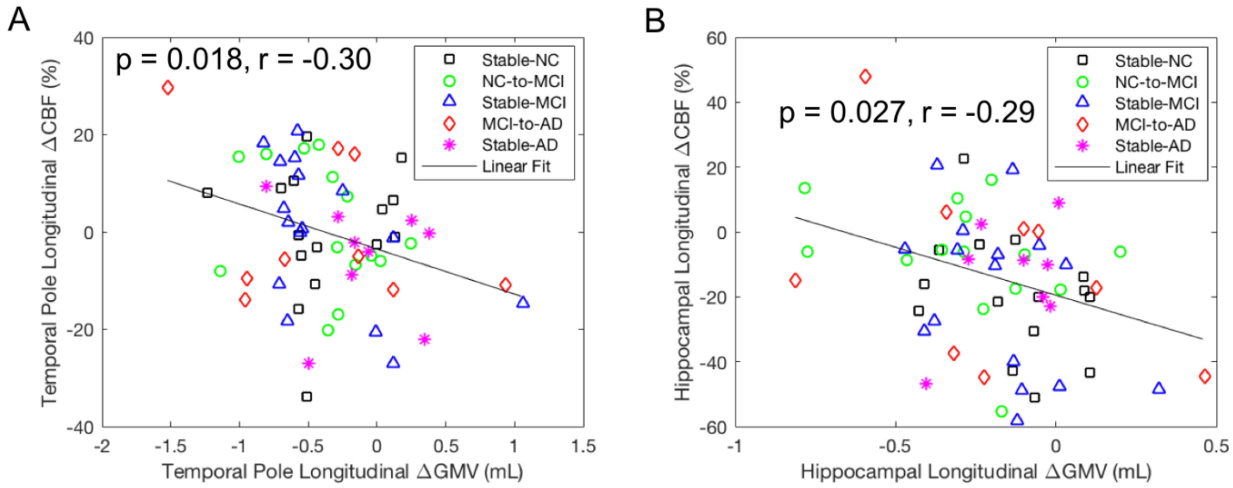

Supplementary Figure 4.. Correlation of longitudinal CBF changes from Time 2 to Time 3 with longitudinal GMV changes from Time 2 to Time 3 after adjusting for age, gender, and time gap for all of the subjects (n = 63). Longitudinal CBF changes were negatively associated with longitudinal changes of GMV values in the same regions: (A) temporal pole and (B) hippocampal regions.

© 2023. Zhou TD et al. Published online at http://www.aginganddisease.org/EN/10.14336/AD.2023.0430
